# Explainable SHAP-space fall risk profiling from electronic health records for inpatient prevention planning

**DOI:** 10.1038/s44401-026-00138-4

**Published:** 2026-09-10

**Authors:** Matthias Schulte-Althoff, Peter Krappen, Felix Bießmann, Sebastian Jäger, Rahel Gubser, Armin Hauss, Felix Balzer, Tim Kilgus, Daniel Fürstenau

**Affiliations:** 1 Department of Information Systems, Freie Universität Berlin, School of Business & Economics, Berlin, Germany; 2 Institute of Medical Informatics, Charité – Universitätsmedizin Berlin, Berlin, Germany; 3 Berlin University of Applied Sciences and Technology (BHT), Berlin, Germany; 4 Einstein Center Digital Future (ECDF), Berlin, Germany; 5 Business Division Nursing, Administrative Department for Academic Health Professions in Practice – Nursing Science and Quality Development, Charité – Universitätsmedizin Berlin, Berlin, Germany; 6 University of Cambridge, Cambridge, UK

**Keywords:** Computational biology and bioinformatics, Health care, Medical research

## Abstract

Falls in hospital settings are common and costly. They happen for many different reasons, which limits the effectiveness of one-size-fits-all prevention strategies. In a retrospective observational study using electronic health record data from a large German university hospital between 2016 and 2022, we trained an ensemble model to predict inpatient falls. We then derived patient-level SHapley Additive exPlanations (SHAP) and clustered model-detected fallers to identify recurrent SHAP-based risk profiles. The resulting centroids were applied to alerted patients in the held-out test set. The final model achieved an area under the receiver operating characteristic curve of 0.95 and an area under the precision-recall curve of 0.36 on the test set, outperforming a baseline model that used only guideline features. We benchmarked SHAP-space clustering against raw feature-space clustering techniques in this alerted test cohort, finding that SHAP-space K-means showed the largest separation in observed fall incidence. The nine clusters identified were then mapped to clinically interpretable risk profiles. These profiles link model alerts to guideline-consistent prevention domains. However, further external validation studies are needed before clinical deployment.

## Introduction

Falls are a major patient-safety concern. Globally, they account for circa 684,000 deaths each year and approximately 37.3 million events requiring medical attention^1^, while 0.85% to 1.5% of total healthcare costs are attributable to falls^2^. In hospitals, falls are, among other consequences, associated with injury, prolonged length of stay, and increased care burden. Although up to 42% of falls may be preventable through nonpharmacological interventions^3^, effective prevention remains difficult because fall risk is multifactorial and heterogeneous across patients. With ageing populations, hospitals are increasingly caring for older, frailer inpatients who have high baseline fall risk and require resource-intensive prevention. At the same time, nursing teams must operationalise multifactorial recommendations under resource constraints, making risk profiling for decision support a health-systems priority.

In routine care, fall risk is commonly assessed using manual checklist-based instruments such as the Morse Fall Scale, STRATIFY, and Hendrich II, which are often complemented by functional assessments such as the Timed Up and Go or Tinetti tests^4–8^. While these tools capture important risk factors, systematic reviews have also shown limited predictive validity for distinguishing high- from low-risk patients and a tendency towards higher sensitivity at the expense of specificity^9–13^. Consequently, many inpatients may be flagged as high risk, which could result in a lack of focus and hinder a more targeted prevention. At the same time, the evidence base on fall risk has broadened to include factors such as diabetes, frailty, pain, and malnutrition^14–17^, reinforcing the need for a more individualised risk assessment.

Recent guidelines have therefore shifted emphasis from a more generic towards personalised screening and prevention. The World Falls Guidelines^18^ recommend that patients identified as high risk should receive a comprehensive multifactorial assessment and tailored interventions aligned with their specific combination of risk factors. Complementary national guidance, such as the German Expert Standard on Falls Prevention in Nursing, similarly emphasises institution-specific assessment and subsequent prevention^19^. Operationalising this recommendation in routine workflows remains a difficult task, as clinicians have to make sense of interacting risks spanning a range of different factors, such as mobility, cognition, and various comorbidities—often under substantial time and staffing constraints^18,20^.

Aiming towards a multifactorial assessment, recent machine-learning studies have used diverse data sources and settings for fall-risk modelling, including acute inpatient Electronic Health Records (EHRs)^21–24^, long-term care data^25^, disease-specific cohorts^26^, sensor-based trajectories^27^, and geriatric assessments^28^. For example, in acute care real-time risk profiling was proposed^23^, whereas other studies illustrated how multi-domain or subgroup-specific modelling can enrich fall-risk assessments^24–26^. Complementary work has used sensor trajectories^27^ or explainability on geriatric assessment features^28^. In a study on inpatient fall prediction using Explainable Artificial Intelligence (AI), it was reported that SHapley Additive exPlanations (SHAP) consistently identified low albumin, impaired transfer ability, and use of sedative-hypnotics or diabetes medications as important contributors to fall risk^29^. These studies show that combining information across clinical domains can improve risk characterisation beyond current tools, while the increasing availability of digital patient records makes such modelling possible at scale^30^. At the same time, clinically useful deployment also requires that model outputs can be translated into recommendations that are sensitive to patient-specific risks as well^31,32^.

Beyond the mere predictive performance, a central challenge is whether heterogeneity among patients who trigger an alert can be exposed by a model. Existing approaches often cluster patients in raw or latent feature space, including transformer-based embeddings derived from EHR data^33–36^. Such representations can be informative, but they do not imply similarity in the mechanisms driving the relevant outcome, such as fall risk. Outcome-guided and explanation-aware clustering approaches address this limitation by incorporating model attributions to derive group structures more closely tied to the modelled risk^37,38^. In this context, SHAP usually provides a principled way to express feature-level contributions to individual predictions^39^. However, prior studies have identified important predictors, individual explanation patterns, embeddings, or patient subgroups^28,34,37,38^, but the step from these outputs to prospectively assignable profiles linked to typical prevention domains remains underdeveloped. This gap matters because explanation formats for clinicians can affect trust and decision behaviour^40^, and because patients at similar predicted risk may still differ substantially in the mechanisms driving that risk.

This challenge is consistent with broader work on heterogeneous treatment effects: both risk drivers and preventive needs can vary substantially across patients, but estimating individual-level effects from electronic health records is difficult in practice^41–45^. A pragmatic alternative is to identify groups of alerted patients who share similar risk patterns and then link those to intervention domains. Such an approach can bridge prediction and intervention while preserving interpretability for clinical users. First studies outside the domain of inpatient falls support finding relevant groups by clustering patients in explanation space, such as SHAP values^46,47^. These studies have shown that clustering on explanation space is useful for recovering latent subtypes that relate to a specific class membership. We aimed to adapt this approach for fall prevention.

**Fig. 1:**
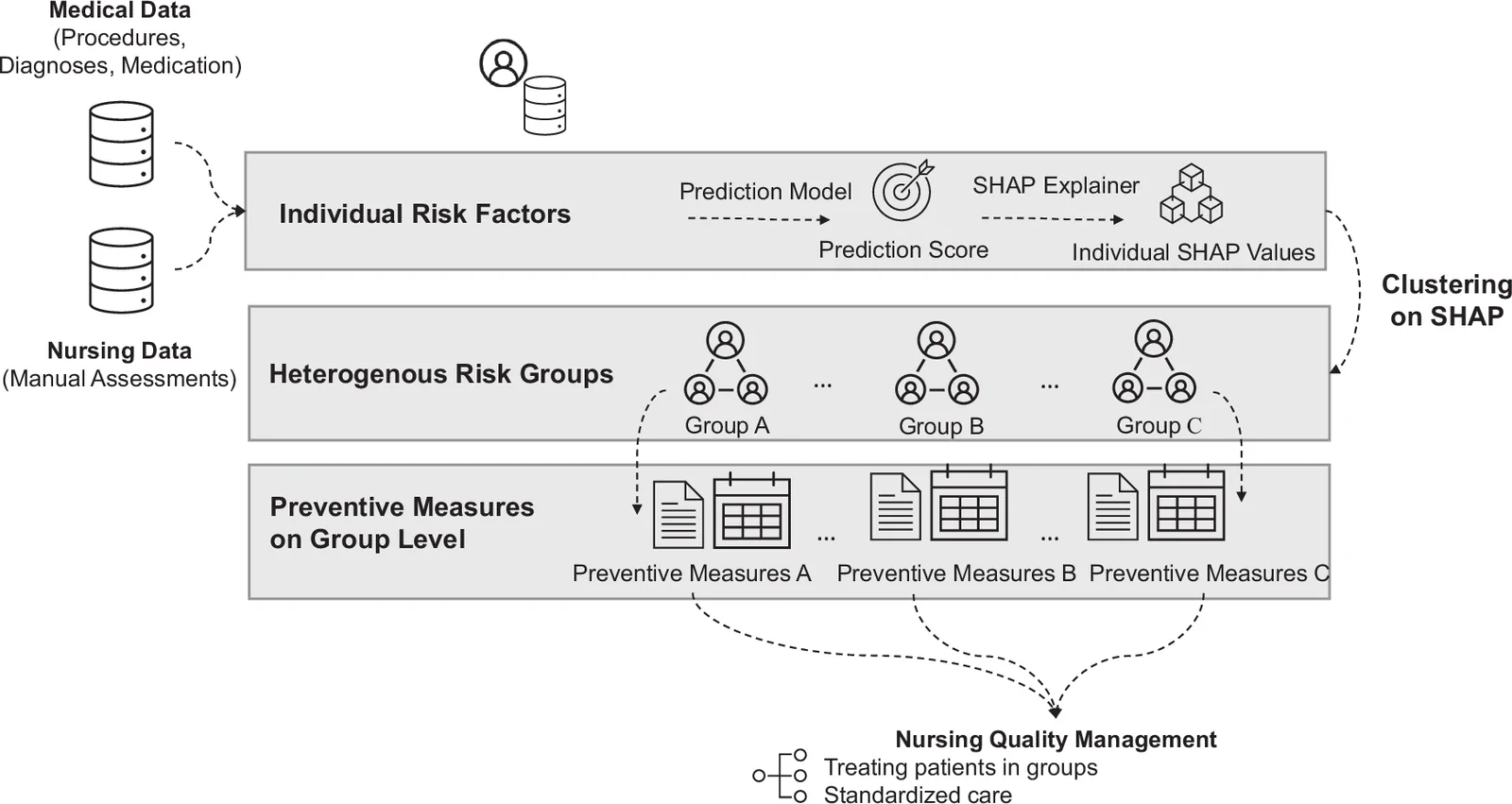
From individual risk factors to group-tailored inpatient fall prevention. Individual electronic health record risk factors are utilised to predict patient-level fall-risk and calculate corresponding SHAP explanations. Clustering in explanation space groups high-risk patients with similar model-attributed risk patterns into clinically interpretable groups. These subgroup profiles can then be mapped to guideline-aligned clinical domains, enabling more targeted inpatient fall-prevention planning than risk scoring alone.

In this retrospective observational study, we evaluate whether explainable fall prediction based on electronic health records can be translated into clinically meaningful group-level risk profiles for fall prevention. Specifically, we train machine-learning models on large-scale hospital electronic health record data, derive patient-level SHAP values, identify SHAP-based profiles among detected fallers in the development cohort, and assess whether these clusters separate the observed fall incidence among alerted patients in the held-out test set more strongly than feature-space clustering techniques. Unlike clustering patient records in the feature space, clustering local explanations groups patients by the features that drove the fall-risk prediction; the resulting profiles are thus linked to the model outcome. Moreover, unlike local explanations considered in isolation, clustering reveals recurrent structure across individual patient profiles. This design links individual risk prediction to reusable subgroup-level profiles and therefore addresses a practical gap between accurate alerts and clinically interpretable prevention planning. The overall modelling idea is shown in Fig. 1.

## Results

### Predictive modelling

Table 1 presents the evaluation metrics selected for the representative models. The L3 weighted ensemble model achieved the highest AUPRC (0.357) on the held-out test set, as well as an AUROC of ~0.946. The primary model also provided substantially better risk stratification than the hospital’s current fall-risk assessment (see Suppl. Table 1). The precision-recall performance exceeded the no-skill baseline, which, given a prevalence of ~1.2%, has an AUPRC of ~0.012.

**Table 1:** Model performance metrics on the test set

ML Metric	Logistic Regression	Neural Net for Classification	RandomForest Gini BAG L2	Weighted Ensemble_L3	LightGBMXT BAG L2	XGBoost: Fall Guidelines
**AUPRC**	0.1079	0.1639	0.3343	**0.3568**	0.3449	0.0486
**AUROC**	0.9044	0.8802	0.9373	0.9463	**0.9472**	0.8225

AUPRC and AUROC metrics for various prediction models to predict falls based on patient history.

The XGBoost: Fall Guidelines model has been trained only on features that are considered in the World Falls Guidelines and a common German fall standard ^18,19^.

Bold values indicate the highest performance for each metric.

In contrast, the simplified model that used only guideline-specified risk factors (26 features including the history of fractures, mobility impairment, cognitive impairment, depression, etc.) performed much worse (AUPRC ~ 0.05, AUROC ~ 0.82). This model, which uses only the guideline-specified features, is intended as a clinically interpretable point of reference and is not intended to be a methodologically equivalent comparator to the full ensemble, as it uses a much narrower feature set. However, the results highlight that additional EHR variables may carry an important predictive signal that the traditional risk factors alone do not capture. For example, the full model identified certain procedure categories, such as complex treatment or physical therapy orders, that were among the top predictors of falls, whereas a model restricted to the features in the guidelines alone might disregard these.

We conducted a paired feature-ablation sensitivity analysis because documentation of nursing care was absent for 76% of patients. The analysis examined whether model performance was driven primarily by documentation status or by actual nursing assessments (Suppl. Tables 4, 5). Eliminating all nursing variables resulted in a decrease in performance to an AUROC of 0.944 and an AUPRC of 0.273. Adding nursing documentation indicators to this model improved the performance by ΔAUROC + 0.00358 (95% CI: 0.00183, 0.00524) and ΔAUPRC + 0.02426 (95% CI: 0.01348, 0.03533). The results suggest that nursing documentation has significant predictive value. However, adding the actual nursing assessment outcomes on top of the documentation indicators yielded further improvements: ΔAUROC = 0.00186 (95% CI: 0.00031, 0.00336) and ΔAUPRC = 0.06744 (95% CI: 0.05463, 0.07982). Thus, while the mere existence of documentation information contributed to the prediction, the actual nursing assessment values added greater performance improvement beyond documentation status alone.

The 24-hour landmark model, as detailed in Suppl. Tables 6, 7, achieved an AUROC 0.798 (95% CI 0.786–0.809) and an AUPRC 0.0529 (95% CI 0.0468–0.0606). The AUPRC was approximately 4.5 times higher than the no-information baseline of 0.0118. Calibration was reasonable, with a calibration slope of 0.869 and a Brier score of 0.0114. At the prespecified threshold *p* ≥ 0.08, sensitivity was 10.2%, specificity 98.8%, Positive Predictive Value (PPV) 9.1%, and Negative Predictive Value (NPV) 98.9%. Compared with the primary full-window patient-level analysis, performance was lower by 0.1485 AUROC and 0.3039 AUPRC. The results show that, although predicting early admission is substantially more difficult, the model still performs better than expected when only information from the first 24 h is used.

The temporal out-of-sample validation, as detailed in Suppl. Tables 8, 9, showed that model discrimination was preserved over time. The primary model here achieved an AUROC 0.953 (95% CI 0.941–0.962) and an AUPRC 0.337 (95% CI 0.286–0.388). Compared to the main model with random patient-wise split, AUROC did not degrade (0.953 vs. 0.946). The AUPRC was slightly lower than in the random split in absolute terms (0.337 vs. 0.357), however with the temporal test-set prevalence being approximately half that of the random test set (0.59% vs. 1.16%). Relative to the baseline defined by the fall prevalence, the temporal AUPRC showed a 57.5-fold lift, compared to a 30.7-fold lift in the random split model. At the prespecified threshold of *p* ≥ 0.08, the temporal test-set had a sensitivity of 55.0%, a specificity of 98.2%, and a PPV of 15.0%. A less conservative sensitivity analysis that assigned patients that appear in different periods to their earliest eligible temporal split yielded similar results, with an AUROC of 0.953, AUPRC of 0.342, and a 58.5-fold lift over the baseline.

### Threshold trade-off analysis

**Fig. 2:**
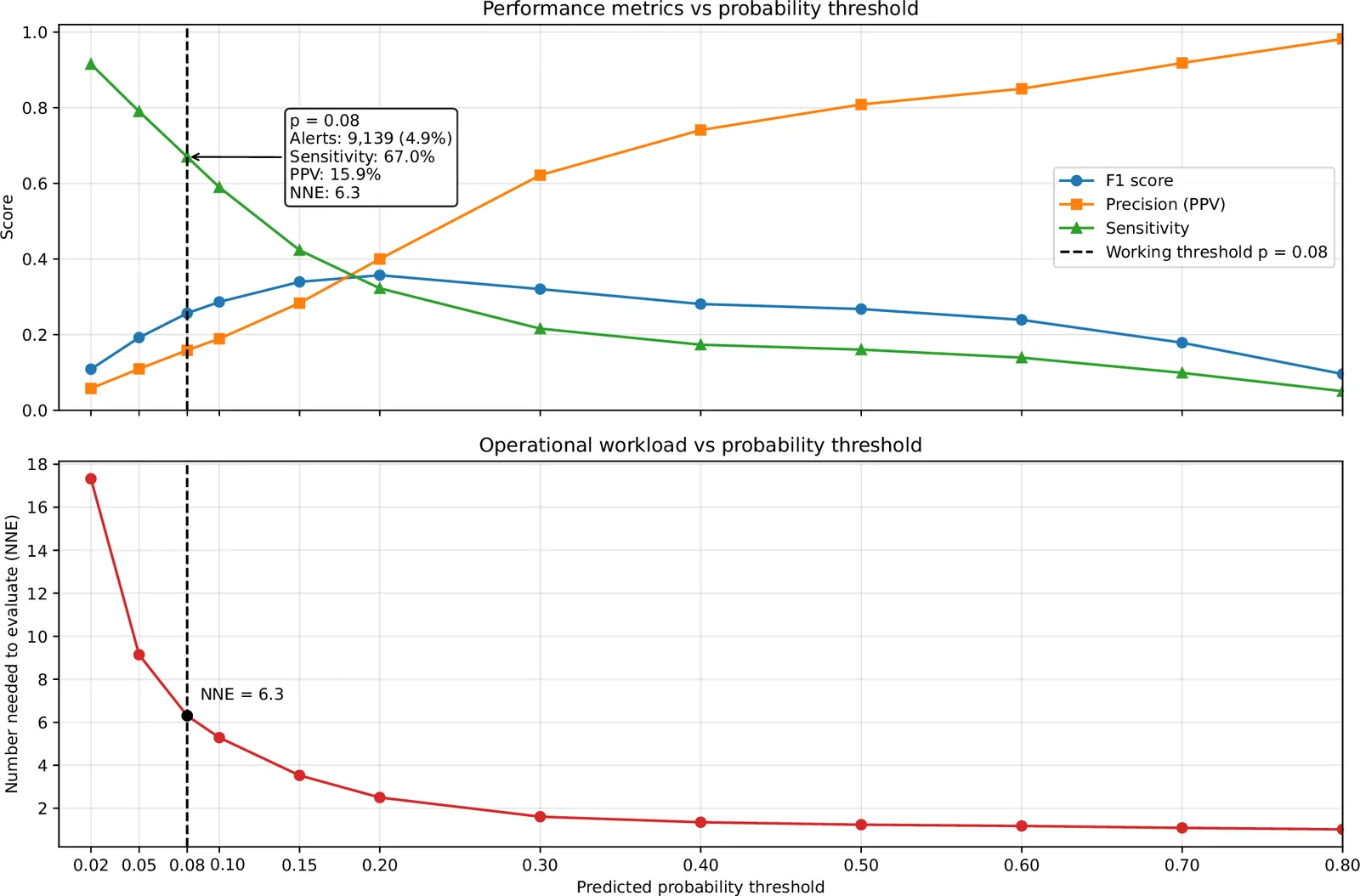
Threshold trade-off analysis. The top figure shows performance metrics at different prediction thresholds. Higher thresholds yield fewer alerts with high precision but miss more falls (low sensitivity). Lowering the threshold increases sensitivity (catching more true fall cases) at the expense of more false positives (lower precision). The chosen operating point should balance the consequences of missed fallers against the review workload and potential burden of additional alerts. The bottom figure shows the *Number Needed to Evaluate NNE* = 1/PPV—that is the number of patients who need to be evaluated to identify one observed faller.

We performed a post-hoc threshold analysis to quantify the trade-off between sensitivity, precision, and operational workload across probability cut-offs (Fig. 2). At the default threshold of 0.50, the weighted ensemble achieved very high specificity (~99.96%) and precision (~81%) with a PPV of 80.9%, but sensitivity was low (~16%), indicating that only a small fraction of fallers would be identified under this conservative setting. This threshold may be too conservative if the clinical objective is to identify a larger proportion of patients who later experience a documented fall.

At the working threshold of *p* ≥ 0.08, the model generated 9139 alerts, corresponding to 4.90% of the held-out test cohort. This operating point captured 1450 of 2164 fallers, yielding sensitivity of 67.0%, PPV of 15.9%, and a number needed to evaluate (NNE) of 6.3. Here, NNE denotes the number of alerted patients who would need clinical review to identify one patient who later had a documented fall in the test set. The final model’s NPV was extremely high (> 99%) due to the rarity of falls. At stricter thresholds, alerts became more precise but far fewer fallers were captured; at more permissive thresholds, more fallers were identified at the cost of a larger number of patients requiring review.

For inpatient falls, the relative costs of false negatives and false positives are asymmetric. In inpatient falls, a false negative—failing to identify a patient who then falls—can lead to injury, extended hospitalisation, and associated costs. False positives—flagging someone who wouldn’t have fallen—may lead to precautionary interventions like closer monitoring, bed alarms, assistance with toileting, etc. Relative to the consequences of a fall, these interventions are generally low-risk and low-cost. Therefore, many hospitals choose a threshold that errs on the side of sensitivity, accepting more false-positive alerts to identify more patients who later fall. In our context, moving from 16% sensitivity to ~67% sensitivity would require about a five-fold increase in evaluations per observed faller identified, with precision dropping from 81% to 16%. Whether this is acceptable depends on resource availability. In the remainder of this paper, threshold adjustments used in our workflow analyses were not applied unless explicitly stated.

### Risk profiles by patient cluster

**Fig. 3:**
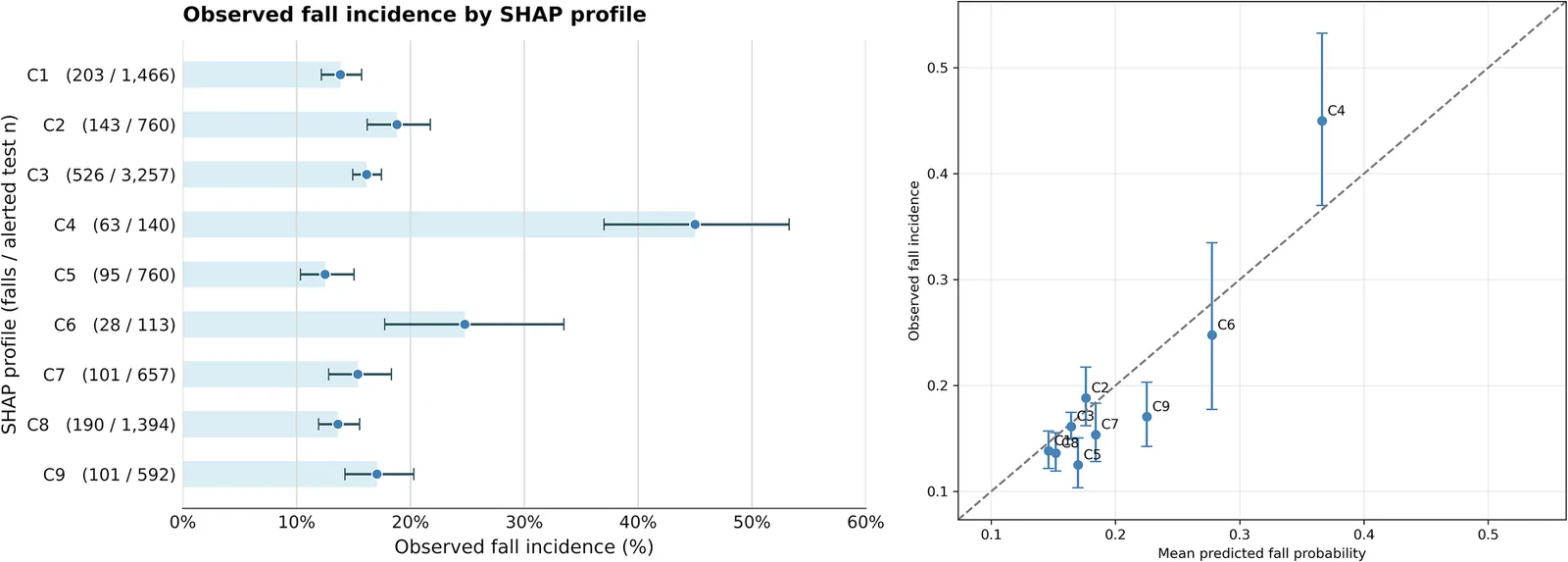
Observed incidence and calibration of SHAP-based risk profiles in the alerted test cohort. **a** Observed fall incidence across the nine SHAP-based risk profiles after applying the fixed *k* = 9 centroids to alerted held-out test patients with predicted fall risk *p* ≥ 0.08. Error bars indicate 95% Wilson confidence intervals. **b** Mean predicted fall probability versus observed fall incidence by profile. Each point in Fig. 3b represents a SHAP-based risk profile after applying the centroids to the alerted test cohort. Error bars show 95% confidence intervals for observed fall incidence. The diagonal line indicates perfect agreement.

For the selected weighted ensemble model, we examined the aggregated local feature importances using SHAP (Supplementary Fig. 3). We then used the signed SHAP values for a K-means clustering as described in the Methods section. We identified nine SHAP-based risk profiles in the discovery cohort. The k = 9 solution was reproducible and met the prespecified viability criteria. Its silhouette score was 0.155, Davies–Bouldin index 1.774, gap statistic 2.501, and mean out-of-bag bootstrap adjusted Rand index 0.856 (95% interval 0.687–0.965). The smallest discovery cluster contained 103 (3.4%) of the 3,016 detected fallers from the development cohort, no discovery cluster was below 2%, and repeat fitting produced identical labels and numerically equal centroids. k = 7 showed higher stability, k = 8 had comparable outcome separation, and some internal metrics continued to improve at higher k. We therefore interpret k = 9 as a clinically useful resolution and not as a uniquely optimal mathematical solution. Higher-k solutions increased fragmentation, with several clusters falling below 5% from k = 11 onward. Figure 3 shows their observed incidence and predicted-versus-observed alignment in the alerted test cohort.

After applying the centroids to the alerted test cohort, the nine profiles differed in terms of both attribution patterns and observed fall incidence. The cluster sizes ranged from 113 to 3257 patients, the observed fall incidence ranged from 12.5% to 45.0%, and the mean predicted fall probability ranged from 14.6% to 36.6%. There was alignment between the predicted risk and the observed incidence on the cluster-level, with Spearman correlation 0.783. The profile with the highest incidence, C4, had an observed fall incidence of 45.0% and a mean predicted fall probability of 36.6%. Other profiles showed similar overall risk but different attribution patterns, as with C1 and C8, for example. Despite the similar incidence and predicted risk, C1 was characterised more strongly by rehabilitation issues, whereas C8 was characterised more strongly by complex treatments and radio- and chemotherapy.

Table 2 summarises the nine SHAP-based risk profiles and the corresponding guideline-aligned clinical review domains. These mappings are intended to provide prompts for further prevention planning, rather than representing empirically validated intervention effects. Importantly, several cluster-defining SHAP drivers are not themselves modifiable, such as age, prior falls, diagnostic procedures, or complex treatment indicators. Special attention should be given to domains that are potentially modifiable or amenable to intervention, such as mobility and transfer safety, cognition or delirium, medication and orthostasis, among others.

**Table 2:** SHAP-based risk-profile summary for the *k* = 9 solution

Profile	Discovery *n* (%)	Alerted test *n*	Falls	Observed incidence	Mean predicted risk	Median age	Male	Dominant SHAP drivers	Guideline-aligned clinical review domains
**C1: Rehabilitation/complex-care**	352 (11.7%)	1,466	203	13.8%	14.6%	75	58.1%	Early rehabilitation/physical therapy (+); computed tomography (+); complex treatment (+); examination/clarification (−)	Rehabilitation and functional-care review: mobility, transfer function, ADL support, environmental safety, patient education (p. 24), physical activity/exercise where appropriate (p. 23), nutrition/vitamin D or bone-health assessment where indicated (p. 18), and medication/orthostasis review if clinically relevant (p. 21–22).
**C2: General Complex Care**	276 (9.2%)	760	143	18.8%	17.6%	75	57.1%	Care-episode/movement marker (+); computed tomography (+); complex treatment (+); supplementary care information (+)	Complex-care and transition review: assess whether ward movement or care-episode complexity reflects instability; review mobility/transfer safety, early mobilisation, orientation, hydration, constipation, feeding assistance, sleep, family involvement (p. 16), caregiver/staff support (p. 19), and orthostatic or pain-medication contributors where relevant (p. 21–22).
**C3: General Care, head-injury**	660 (21.9%)	3,257	526	16.1%	16.4%	78	51.6%	Supplementary care information (+); computed tomography (+); early rehabilitation/physical therapy (+); head injury (+)	Cognitive/neurological review: assess head-injury or neurological status, cognition/delirium/orientation, supervision needs, mobility/balance, transfer assistance, rehabilitation needs, cognitive-behavioural or occupational-therapy domains where relevant (p. 12), nutrition/vitamin D and alcohol/substance-use assessment where relevant (p. 18), and patient education (p. 24).
**C4: Care-episode/CT profile**	171 (5.7%)	140	63	45.0%	36.6%	73	54.3%	Care-episode/movement marker (+); computed tomography (+); supplementary care information (+); complex treatment (+)	Enhanced multidisciplinary review because of high observed incidence and small cluster size: acute diagnostic context, mobility/transfer safety, supervision and toileting plan, treatment burden, early mobilisation/orientation/hydration/constipation/nutrition support (p. 16), ADL/caregiver support (p. 19), orthostatic review (p. 21), pain or medication review where relevant (p. 22), and patient education (p. 24).
**C5: CT, prior-fall/ circulatory-support**	276 (9.2%)	760	95	12.5%	17.0%	71	60.3%	Computed tomography (+); care-episode/movement marker (+); prior-fall documentation (+); circulatory measures (+)	Prior-fall and circulatory/cardiovascular review: fall history, mobility/transfer safety, orthostatic blood pressure, syncope or cardiovascular contributors, circulatory procedures/support, hydration, compression or pharmacological options where appropriate (p. 21), ADL/general-activity review (p. 19), patient education (p. 24), and medication review targeted to cardiovascular or pain-related contributors (p. 21–22).
**C6: High-attribution supplementary-care/CT**	103 (3.4%)	113	28	24.8%	27.8%	75	48.7%	Supplementary care information (+); computed tomography (+); complex treatment (+); care-episode/movement marker (+)	High-dependency complex-care review: supplementary-care needs, acute diagnostic/treatment burden, supervision, early mobilisation, orientation, hydration, constipation, feeding assistance, sleep and family involvement (p. 16), balance/strength/walking-oriented training where appropriate (p. 12), physical activity/exercise (p. 23), nutrition/vitamin D or bone-health assessment (p. 18), and caregiver/staff support (p. 19).
**C7: Behavioural/mental-health**	333 (11.0%)	657	101	15.4%	18.4%	72	44.1%	Mental, psychosomatic and behavioural disorders (+); supplementary care information (+); early rehabilitation/physical therapy (+); complex treatment (+)	Nutritional assessment (adequate vitamin D intake; serum 25 OH vitamin D levels), assessment of substance abuse and excessive alcohol intake (p. 18), Physical exercise (in community settings; Pilates, Yoga) (p.23), Behavioural therapy (for mild cognitive impairment/ dementia: group exercise. cognitive behavioural therapy and Occupational therapy) (p.12), Rehabilitation and fall-history review: prior falls, mobility/balance, transfer assistance, ADL support, environmental hazards, patient education (p. 24), balance/strength/walking training where appropriate (p. 12), physical activity/exercise (p. 23)
**C8: Oncology/complex-treatment**	280 (9.3%)	1,394	190	13.6%	15.2%	70	61.9%	Complex treatment (+); computed tomography (+); radiotherapy/nuclear medicine therapy/chemotherapy (+); care-episode/movement marker (+)	Treatment-related vulnerability review: fatigue, weakness, anaemia, neuropathy, pain, nausea, deconditioning, mobility/transfer safety, hydration/nutrition, physiotherapy or supervised activity, pain assessment and management (p. 17, p. 22), medication review for pain or neuropathic-pain treatments (p. 22), non-pharmacological approaches such as physiotherapy where appropriate (p. 22), nutrition/vitamin D or bone-health assessment (p. 18), and early mobilisation/orientation support in complex care (p. 16).
**C9: Prior-fall/complex-care** (weak attribution)	565 (18.7%)	592	101	17.1%	22.5%	66	52.0%	Examination/clarification (−); prior-fall documentation (+); complex treatment (+); hypertension (−)	Individual SHAP-guided review rather than a strong profile template. Check prior falls, mobility/transfer safety, environmental hazards, continence/toileting, orthostasis or cardiovascular contributors where relevant (p. 21), pain or medication contributors where relevant (p. 22), patient education (p. 24), ADL/general-activity review (p. 19), physical activity/exercise where appropriate (p. 23), and nutrition/vitamin D or bone-health assessment where indicated (p. 18).

Discovery size refers to the discovery cohort sample of 3016 fallers detected by the model from the development cohort used to learn the *k* = 9 profile centroids. Alerted test size, falls, observed incidence, and mean predicted risk refer to the alerted test cohort with predicted risk *p* ≥ 0.08 after applying the centroids. SHAP drivers are signed model-attribution drivers; positive values increase predicted fall risk and negative values decrease predicted fall risk relative to the model baseline. The final column suggests interventions prompted for clinical review with regard to the clusters’ risk profile based upon the World Falls Guidelines^18^.

*ADL* Activities of Daily Living, *CT* to computed tomography.

For assignment of newly alerted patients to an existing SHAP profile, we used nearest-centroid mapping based on the discovered *k* = 9 centroids. A patient was assigned to the nearest centroid only if the Euclidean distance to that centroid was no greater than the 90th percentile of within-cluster distances estimated in the discovery cohort. Patients exceeding this threshold were treated as low-confidence matches and left unassigned; for such patients, the nearest candidate profiles should be displayed for clinical review. In internal feasibility analysis, this distance-based rule assigned 93% of alerted test-cohort patients to one of the nine profiles.

### Outcome separation benchmarking of clustering approaches

Across clustering methods, SHAP-space K-means produced the widest observed-incidence range and the highest incidence ratio among the clustering-based approaches evaluated in the alerted test cohort (Table 3). Observed fall incidence across SHAP-space profiles ranged from 12.5% to 45.0% (range 0.325; ratio 3.60; IQR 0.050), with positive alignment between cluster-level mean predicted risk and observed incidence (Spearman rho = 0.783; weighted cluster-level calibration mean absolute error (MAE) = 0.017). Feature-space clustering methods produced narrower incidence ranges (0.070–0.111) and lower incidence ratios (1.51–1.85), indicating weaker outcome stratification among alerted patients. These findings suggest that clustering in explanation space yields interpretable attribution profiles with stronger observed-incidence separation than the feature-space clustering comparators evaluated here.

**Table 3:** Outcome-separation benchmark in the alerted test cohort

Method	k _effective_	Included Alerted-test *n*	Falls	Incidence range	Max/min incidence ratio	Incidence IQR
**SHAP-space K-means**	**9**	**9139**	**1450**	**0.325**	**3.60**	**0.050**
Raw K-means	8	9102	1449	0.087	1.65	0.021
Raw GMM	3	8969	1389	0.111	1.77	0.056
Raw Ward	6	9100	1450	0.090	1.85	0.041
Raw Spectral	7	9018	1432	0.072	1.63	0.040
PCA(95%) + K-means	6	9115	1450	0.070	1.51	0.044

SHAP-space K-means and feature-space comparators were learned in the discovery cohort and then applied to held-out test-patients with predicted fall risk *p* ≥ 0.08.

Metrics summarise the distribution of observed fall incidence across groups in the alerted test cohort. Clusters contributing < 1% of the evaluation cohort were excluded from method-level separation metrics; thus *k*_*effective*_ may be < 9. These small clusters are reported in Suppl. Table 13. Higher values indicate stronger observed-incidence dispersion for range, ratio, IQR.

Bold values indicate the highest performance for each metric.

## Discussion

Using a large-scale EHR from 2016 to 2022 with 932,102 patients, the weighted ensemble model achieved AUROC ≈ 0.95 and AUPRC ≈ 0.36 on a held-out test set, substantially outperforming a guideline-features-only model (AUROC ≈ 0.82; AUPRC ≈ 0.05). The model enabled two clinically relevant analyses. First, threshold analysis made the trade-off between sensitivity and precision explicit: at *p* ≥ 0.08, 4.90% of held-out test patients triggered an alert, 67.0% of fallers were identified, and 6.3 alerted patients required review to identify one patient who later had a documented fall. Second, clustering signed SHAP explanations identified nine risk profiles. After applying the fixed centroids to the alerted test cohort, observed fall incidence varied from 12.5% to 45.0% across profiles, and SHAP-space clustering separated observed incidence more strongly than the evaluated feature-space comparators. These findings suggest that explanation-based profiling can add a clinically interpretable layer between risk prediction and multifactorial fall-prevention.

The predictive results compare favourably with prior fall-prediction studies, although direct numerical comparisons should be interpreted cautiously because of different study designs. Previous work has demonstrated the value of machine learning across several fall-risk prediction settings^21,22,25,26,48^. Explainability has been used for fall-risk modelling, including work based on gait and geriatric assessments in older adults^28^ and recent inpatient EHR-based prediction using SHAP to identify patient-level risks^29^. Our study extends this line of work, as the main contribution is not only higher discrimination, but the conversion of individual local explanations into prospectively assignable group profiles. In this respect, the study also builds on explanation-space clustering approaches used for subtype discovery and risk stratification in other biomedical settings^46,47^, while applying them to the implementation problem of inpatient fall prevention.

The threshold analysis is important because fall-prevention workflows are constrained by time, alert burden, as well as the consequences of missed falls. This concern is consistent with work on fall-prevention alarms, which has emphasised that indiscriminate alerting can create low-value workload^13^. Lowering the threshold increased sensitivity but also increased the number of patients requiring review; stricter thresholds improved PPV but missed a larger proportion of fallers. This kind of data-driven planning is not as easily possible with checklist scores that typically yield point totals rather than calibrated probabilities and do not support unit-specific probability cut-offs^4–6,9^.

The SHAP-profile results address a second implementation challenge: alerted patients are not clinically homogeneous. The emphasis on personalised fall risk assessment is consistent with several scientific findings^49–51^ and the World Falls Guidelines assertion of the efficacy of tailored interventions^18^. Additionally, the literature suggests that clustering can help to summarise patient heterogeneity into interpretable groups when used cautiously^34^. Explainable AI has been proposed as a way to make AI-supported clinical decisions more transparent and actionable^52^, but the implementation challenge is to integrate isolated explanations as outputs that also fit clinical workflows. In our setting, patients could have similar predicted risks for different model-attributed reasons, and those differences matter for prevention review. This distinction can be illustrated by two adjacent profiles in our analysis. C1 and C8 had almost identical observed fall incidence and mean predicted risk, but differed in their dominant model-attributed drivers of fall risk: C1 was more strongly characterised by early rehabilitation and functional-care markers, whereas C8 was more strongly characterised by complex treatment and oncology-associated therapy. In a future decision-support workflow, both patients would warrant clinical review because of elevated risk, but the profile prompt could direct attention to different review domains: mobility and rehabilitation needs in the former case, and treatment-related vulnerability, fatigue, or neuropathy, among others, in the latter. Thus, the practical advantage of explanation-space profiling over risk scores is that it may reveal recurrent reasons for model predictions^46,47^—reasons that can then be translated into domains that clinicians can review.

A major impetus for this work was to make the recommendations of the World Falls Guidelines to be more operationalisable in a hospital setting. The World Falls Guidelines call for multifactorial falls risk assessments and personalised interventions for those at high risk^18^. The Registered Nurses’ Association of Ontario (RNAO) guideline gives further guidance in categorising fall-risk factors as non-modifiable and potentially modifiable or intervention-amenable^53^. Some dominant SHAP drivers, such as age, prior fall documentation, or oncological therapy are not intervention targets and should not be read causally. Instead, they can function as markers that direct clinicians towards review domains such as mobility and transfer safety, cognition or delirium, and medication. In future decision support, a possible use of these profiles would be a prompt for clinician assessment, combined with the patient’s individual SHAP explanation.

The additional sensitivity analyses clarify the intended scope of the model. The primary model used feature windows at the patient level that differed between fallers and non-fallers. Fallers contributed information up to their first fall, and non-fallers up to the end of the observation period. While this design supports retrospective risk profiling, it does not mirror an admission-time alerting scenario. In the 24 h landmark analysis, which used only information available within the first day of admission and predicted post-landmark falls at the admission level, performance was substantially lower while remaining circa 4.5 times above the prevalence baseline. These results suggest that early prediction over a fixed horizon is a more demanding task, and that part of the primary model’s performance reflects information accumulated during the inpatient stay. Conversely, a sensitivity analysis for temporal validation in the 2022 test cohort showed preserved discrimination, supporting robustness to temporal changes.

This study has several limitations. It was retrospective and conducted at a single hospital, so documentation practices, case mix, and model performance may differ across institutions. We did not include all potentially relevant data streams, such as medication administration records or real-time physiological measures, which may further improve prediction. The sampling design included clinical services, in which falls were prevalent with ICUs being excluded. Therefore, the observed prevalence and estimated fall prediction should not be interpreted as hospital-wide estimates. In addition, the primary patient-level model used asymmetric feature windows: fallers contributed information up to the first fall, whereas for non-fallers information up to discharge was used. The 24 h landmark analysis shows that early fixed-horizon prediction is substantially more difficult, indicating that part of the primary model’s performance reflects information accumulated during the hospital stay.

Further limitations include that the risk profiles were derived in a discovery cohort from the development cohort and then evaluated internally on a test cohort of alerted patients. Although the prediction model can generate risk scores and patient-level explanations immediately, prospective profile assignment requires stable centroids and assignment thresholds, either established during local model development or updated during a monitored warm-up period. The k-sensitivity analysis showed that *k* = 9 was reproducible and viable, but not uniquely optimal. Some profiles were adjacent in SHAP space and should be understood as related profiles rather than distinct clinical groups. Furthermore, although SHAP values quantify the drivers of model-attributed predictions, they should not be interpreted as causal treatment targets. Finally, the intervention mapping was theoretical in nature and based on guidelines. While the World Falls Guidelines support both multifactorial assessment and the provision of tailored interventions for high-risk patients, our study did not empirically test whether the retrieved prevention templates actually improve clinical outcomes.

Despite these limitations, our findings suggest that EHR-based fall-risk profiling can be used to translate predictions into clinically interpretable prompts for intervention. By linking alerts to dominant risk factors and group-level assessments, this approach may allow for a more targeted review of multifactorial fall risk than broad, checklist-based screening alone. Further external validation studies are required to test the transportability of the results, their effect on workflows, and whether profile-guided prevention reduces falls or improves resource allocation in routine care.

## Methods

### Data sources and coding

We conducted a retrospective observational study using pseudonymised EHR data from a large German university hospital. We considered inpatient admissions from 2016 to 2022. All patients who experienced one or more falls during this period were included, as well as a sample of patients who did not fall. Inclusion was limited to adults (age ≥ 18), and to ensure broad representation across hospital services, non-fallers were drawn from the 20 departments with the highest fall counts (e.g. oncology, geriatrics, neurology, neurosurgery, internal medicine and psychiatry) (In analyses restricted to admissions with available hospital fall-risk assessment scores, as detailed in Supplementary Table 1, based on logistic regression, predicted fall probability increased from approximately 0.46% for score “0” to 2.57% for “+” (increased fall risk) and 7.00% for “++” (strongly increased fall risk). Pairwise comparisons implied by the fitted model indicated that, relative to score “0”, the odds of a documented fall were approximately 5.65-fold higher for “+” and 16.13-fold higher for “++”). Intensive care units were excluded because patient mobility, sedation and fall prevention workflows differ substantially from those in general inpatient wards. This sampling strategy focused on services where fall prevention is a routine operational issue.

The final dataset comprised 932,102 unique patients, of whom 10,443 (1.12%) had at least one recorded fall during their hospital stay. Consequently, the dataset was highly imbalanced (see Table 4). We addressed the class imbalance by selecting appropriate evaluation metrics, such as AUPRC. For each patient, we extracted the following data: demographics (age and sex); diagnoses; procedures; and nursing care data (In some cases, diagnoses may not have been coded until the end of the hospital stay. In these cases, the diagnosis date is too late and is sometimes disregarded for machine learning. However, procedures necessary for billing purposes were subject to more precise dating.), which encompasses, among others, mobility, gait, medication, and fall risk assessment scores documented by nursing staff at admission and every seven days thereafter (the hospital uses a three-level scale: “0” = no risk, “+” = some risk, “++” = high risk). Sex was extracted from the hospital administrative record as a binary recorded variable (female/male) with 190 missing values. No gender identity variable was available in the source systems.

**Table 4:** Patient attributes and faller vs. non-faller comparison from 2016 to 2022

Characteristic	All Patients (*n* = 932,102, 100%)	Non-Faller (*n* = 921,659, 98.8%)	Faller (*n* = 10,443, 1.12%)
**Recorded sex male**	435,324 (46.71%)	429,578 (46.62%)	5746 (55.02%)
**Recorded sex female**	496,588 (53.29%)	491,891 (53.38%)	4697 (44.98%)
**Age (years)**	57.44 ± 19.76	57.23 ± 19.70	76.03 ± 15.72
**Diagnoses/patient**	4.82 ± 6.73	4.67 ± 6.48	17.96 ± 12.46
**Procedures/patient**	1.96 ± 3.46	1.88 ± 3.34	8.90 ± 6.12

Values are presented as mean ± standard deviation or count (percentage). 190 patients with unknown sex in the all-patient/non-faller columns.

Diagnosis data included ~12.18 million entries coded in ICD-10-GM (the German modification of the International Classification of Diseases, 10th Revision). Procedure data included ~5.72 million entries coded in OPS (Operationen- und Prozedurenschlüssel, the German procedure coding system). Both coding systems are hierarchical, allowing aggregation into broader categories when needed. Based on clinical consultation, we applied a data cleaning step: diagnoses marked with low certainty (about 4.1% of diagnosis entries) were removed to avoid spurious information. Missing age data for a small fraction of non-fallers (<0.05%) were imputed with the median age (57 years), with no age data missing for fallers. Nursing care data were absent in 76% of all patients, primarily because routine nursing documentation was not initiated for patients who were screened at admission as sufficiently independent/fit and therefore did not require a formal nursing care assessment within the nursing care module. We also derived from hospital records whether each patient had any documented falls prior to the first inpatient fall (for fallers) or during their hospital stays (for non-fallers), and we recorded the initial nursing risk assessment score for falls at admission. These serve as baseline comparisons for our model’s predictive performance. To prevent data leakage, only data available before a fall incident or before discharge for non-fallers was used via the clinic’s timestamps.

Fallers were significantly older (Cohen’s d = 0.956) and had more complex medical histories. In our data, the five most common diagnosis categories among fallers were forms of heart disease, metabolic disorders, potential health risks based on family or personal history, hypertension, and diseases of the muscles (see Suppl. Tables 2, 3). Non-fallers shared many of the top diagnoses (reflecting overall hospital case mix), but fallers had higher rates of cardiovascular and metabolic issues, likely due to their higher age. On the procedure side, fallers most frequently underwent imaging, circulatory system procedures, e.g., catheterisations, and oncological treatments, such as radiation or chemotherapy.

### Predictive modelling

**Fig. 4:**
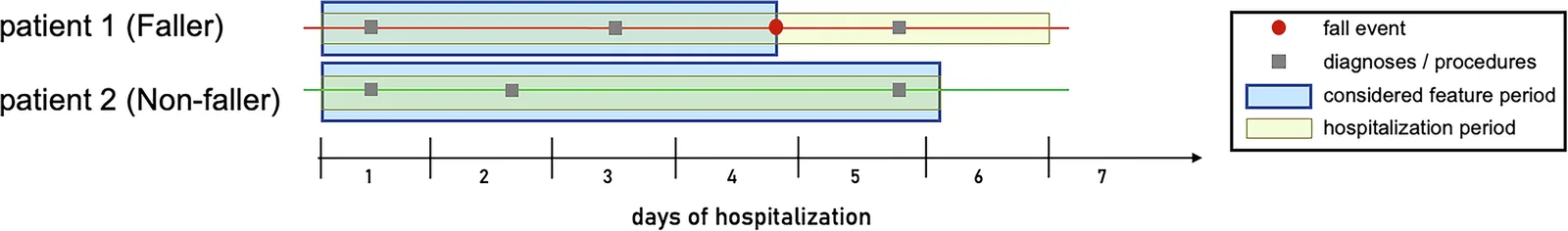
Construction of patient-specific feature windows during hospitalisation. For fallers, only diagnoses and procedures recorded before the first fall event were included in the predictor window to avoid leakage from post-event information. For non-fallers, features were accumulated until discharge. The schematic illustrates the different temporal cutoffs used to construct model inputs.

We formulated fall prediction as a binary classification problem at the patient level: fall vs. no-fall during the hospital stay. Multiple inpatient records belonging to the same patient were mapped to a single patient identifier and aggregated into one feature vector. The train/test split was performed at the patient level to avoid patients contributing to both model development and held-out evaluation. Features were constructed from the patient’s data available up to the time of fall for fallers, or up to the end of the hospital stay for non-fallers (see Fig. 4). In practice, for patients who fell, we included all diagnoses and procedures recorded before their first fall event to avoid labelling consequences of the fall as causes. Guideline-specified fall risk factors included: walking impairment; balance/gait impairment; use of a walking aid; gait assessment; fall within the last 12 months; number of falls in the last 12 months; transfer impairment; transfer assistance; cognitive impairment; disorientation (time/place/own person); agitation; confusion; bed-mobility impairment; bed-mobility assessment; skin condition relevant to mobility; presence of medical items (e.g., infusion, catheter); impairment of excretions; urinary/faecal incontinence; nocturia; use of psychotropic or sedative medications; and decubitus. For patients who did not fall, we included data from their entire hospitalisation. Each patient thus has a feature vector summarising their demographics, diagnoses, procedures, and nursing care data prior to fall or prior to discharge for non-fallers. Thus, the primary analysis should be interpreted as a cumulative patient-level prediction task over the observation period and not admission-specific. Categorical features (ICD-10-GM and OPS) were one-hot encoded using codes on the group level. A higher number of digits indicates a more specific diagnosis, e.g., “J45” indicates asthma, while “J45.0” indicates predominantly allergic asthma. Like the ICD, OPS codes have a hierarchical structure, e.g., “8–98” indicates various complex treatments, while “8–981” indicates a neurological complex treatment. The first two characters of the codes were used for grouping. We built both a broader EHR model containing all of the previous features and a smaller reference model restricted to the guideline-specified fall risk factors.

We leveraged an AutoML library (AutoGluon) to explore a suite of models—logistic regression, multilayer perceptron (neural network), random forest, gradient-boosted trees (XGBoost and LightGBM), and stacked ensembles. We performed a patient-wise random split into a training set used for cross-validation, model selection, and profile discovery (80%) and a final held-out test set for reported performance, threshold analysis, and profile evaluation (20%). Model selection and hyperparameter tuning were performed using 5-fold cross-validation on the training set, with the area under the precision–recall curve (AUPRC) as the primary metric for optimisation. AUPRC is well-suited for imbalanced data because it focuses on performance in the minority class and is less dominated by the abundance of negative cases. It also evaluates model performance across all possible prediction thresholds, aligning with our interest in how different threshold choices affect precision and recall. For comparability, we additionally report operating-point metrics (sensitivity/recall, specificity, precision/PPV) at selected probability thresholds.

To address severe class imbalance, we optimised model selection using AUPRC and evaluated all final results on the held-out test set with the natural outcome distribution. We avoided using oversampling techniques, such as Synthetic Minority Over-sampling Technique (SMOTE), during training to keep the training data as representative as possible. To improve the understanding of the model predictions and to investigate the underlying fall risk factors in detail, we used SHAP, a game-theoretic technique that can interpret model outcomes^39^. The resulting SHAP values indicate the mean deviation from the mean prediction when conditioning on a single feature, thereby revealing the impact of individual features on model outcomes. Positive SHAP values increase the predicted probability of falling relative to the model baseline, whereas negative SHAP values decrease it. By relating the magnitude of features to their relevance in the model predictions, SHAP values can provide insight into different risk factors and latent interactions at the instance level^54^.

Because a large proportion of patients lacked nursing care documentation, we conducted a missingness sensitivity analysis to distinguish signal from the presence of documentation from actual nursing assessments (Suppl. Tables 4, 5). We compared four models using the same train/test split as the primary analysis but refit leakage-controlled models under four feature configurations: (i) the full model including nursing variables, (ii) a model excluding all nursing variables, (iii) a model excluding nursing values but adding binary indicators for whether a nursing variable was documented, and (iv) a model using only demographic data and nursing documentation indicators. We evaluated performance on the unchanged held-out test set using AUROC and AUPRC. We estimated incremental performance differences with 2000 paired bootstrap resamples at the patient level. This design enabled us to first estimate the incremental contribution of documentation status and second, the incremental contribution of substantive nursing features.

To address the possibility that the primary patient-level model relied on asymmetric information, we conducted a sensitivity analysis of 24 h admissions. Unlike the primary analysis, this one used inpatient admissions as the unit of analysis. We included adults 24 after admission. Admissions ending before or at the landmark were excluded. For patients who fell, the outcome was defined as the first documented inpatient fall after admission plus 24 h and before discharge. Feature construction was restricted to information available at or before 24 h after admission. Model development used the same AutoGluon framework and average-precision optimisation as the primary analysis. Train/test splitting was performed at the patient level to prevent admissions from the same patient from appearing in both the training and test sets. Performance was evaluated on the held-out test set using AUROC, AUPRC, calibration slope, Brier score, and operating-point metrics at the prespecified clinical threshold of *p* ≥ 0.08. Confidence intervals for the AUROC and AUPRC were estimated using 2000 patient-level bootstrap resamples. The resulting landmark cohort included 594,737 admissions from 324,308 patients and 6733 falls. Of those, a total of 1329 occurred at or before the 24 h landmark and were excluded because they were not predictable at that time. The train/test split was performed at the patient level as the primary analysis; therefore, admissions from the same patient could not appear in both model development and held-out evaluation.

To evaluate the robustness of the model with regard to temporal changes, such as changes in documentation practices, we performed a temporal out-of-sample validation. Patients were indexed by admission date. Patients indexed in 2016–2020 were used for model development; those indexed in 2021 were used for temporal validation and probability calibration; and those indexed in 2022 were used as the final test cohort. We again used AutoGluon for model development with AUPRC as the optimisation metric. Probability calibration was performed using Platt scaling on the 2021 temporal validation predictions.

In the primary temporal analysis, patients with records spanning more than one temporal period were excluded to avoid leakage across split boundaries. This conservative approach excluded 110,710 cross-period patients, including 2,327 fallers. A less conservative sensitivity analysis was also conducted in which cross-period patients were assigned to their earliest eligible temporal split rather than being excluded. We evaluated performance in the 2022 temporal test set using AUROC, AUPRC, calibration slope, Brier score, and operating-point metrics at the prespecified threshold of *p* ≥ 0.08. Confidence intervals for the AUROC and AUPRC were estimated using 2000 patient-level bootstrap resamples.

### Threshold trade-off analysis

The envisioned use of our model is as a clinical decision support tool that runs in parallel with standard care processes. During inpatient care, the model would generate a fall risk prediction (with a probability score). If the predicted risk is above a chosen threshold (see Threshold Analysis below), the patient is flagged as high-risk for falls. At that point, the system provides two levels of explanation: (1) an individual SHAP explanation highlighting the patient’s top risk-increasing and risk-decreasing factors, and (2) a cluster assignment suggestion indicating if the patient matches one of the pre-identified risk clusters. Figure 1 illustrates this integration conceptually. In practice, a clinician reviewing the alert would see something like: “High fall risk predicted. Key factors: age 85 (+), recent surgery (+), use of sedative medications (+), independent mobility (−). Patient matches the rehabilitation/complex-care profile.” The clinician can then confirm the relevance of this profile and initiate a tailored care plan accordingly.

A practical aspect is choosing the probability threshold at which to classify a patient as “high risk.” In training, we optimised for overall AUPRC, considering all possible thresholds simultaneously. Depending on hospital priorities, one might prefer a sensitive or a more specific model. For deployment, the model outputs a fall-risk probability score that can be converted into an alert via a probability threshold. We therefore conducted a post-hoc threshold analysis on the held-out test set using candidate thresholds and reporting sensitivity, specificity, precision (PPV), and the number needed to evaluate (NNE = 1/PPV). NNE therefore describes retrospective alert yield and review workload.

### SHAP-based patient clustering

We used SHAP values to identify model-attributed risk patterns among patients whose fall risk was correctly identified by the model. Clustering in SHAP space groups patients by the model-attributed drivers of risk (e.g., mobility, cognition, medications), rather than by feature similarity alone, yielding profiles that are interpretable at the group-level. We used a two-stage profile-discovery and profile-evaluation design. First, signed SHAP vectors were generated for the discovery cohort, defined as detected fallers in the development cohort. Detected fallers were defined using the development-set model predictions at the prespecified threshold of p ≥ 0.08. To keep SHAP computation tractable while retaining a sufficiently large case-only profile-discovery set, we drew a random sample of 3,016 model-detected fallers. The aim of this step was to learn SHAP-attribution patterns among fallers that the model was able to identify. These SHAP vectors were used to estimate the profile centroids in the discovery cohort based on internal clustering metrics. Second, the centroids were applied to the alerted test cohort, defined as held-out test-set patients with the predicted fall risk threshold. Cluster-level sizes, observed fall incidence, mean predicted risk, and outcome-separation metrics were estimated in this alerted test cohort.

We constructed a vector of SHAP values for each patient: *Sp* = (*s*₁, *s*₂, …, *s*_m_), where *m* is the number of features. Here *s*ᵢ is the SHAP value for feature *i*, representing how much that feature pushed the model’s prediction for patient *p* above or below the baseline fall probability. Patients who have similar SHAP value profiles can be considered to have similar risk factor contributions, even if their raw feature values differ. For clustering, we used signed SHAP values instead of absolute ones because they preserve information about whether a feature increases or decreases the predicted fall probability. Since raw SHAP values are already on the positive-class probability scale, no further preprocessing was done to avoid overemphasising noisy features. We applied K-means clustering to the signed SHAP matrix in the discovery cohort.

To evaluate the number of clusters, we compared k = 4 through k = 14 using a number of different cluster evaluation metrics in the discovery cohort: silhouette score, Davies–Bouldin index, Calinski–Harabasz score, gap statistic, and bootstrap stability. Bootstrap stability was assessed by refitting the clustering solution in 100 bootstrap resamples and comparing assignments using the adjusted Rand index. We specified a viable solution as one with minimum discovery-cluster size of at least 2%, mean bootstrap adjusted Rand index of at least 0.75, and silhouette score within 0.03 of the best candidate in the clinically plausible k = 7–9 range. The selected k = 9 solution met these criteria. We also evaluated lower-k mappings to identify adjacent profiles that might represent nested or related profiles. After clustering, we characterised each cluster both externally by patient attributes and in terms of their respective risk factors. Observed-incidence separation and predicted-versus-observed alignment were evaluated only after applying the fixed centroids to the alerted held-out test cohort.

We evaluated whether explanation-space clustering yields risk profiles that are (i) coherent in terms of risk drivers and (ii) clinically meaningful in terms of fall incidence separation among patients who would trigger an alert. SHAP-space K-means was benchmarked against representative feature-space clustering baselines: raw-feature K-means, Gaussian mixture model, Ward agglomerative clustering, spectral clustering, and Principal Component Analysis (PCA, 95%) followed by K-means. All feature-space comparators used k = 9 clusters and were fitted exclusively in the discovery cohort. K-means assigned test episodes to the nearest fitted centroid, whereas the full-covariance Gaussian mixture model (GMM) used maximum posterior component probability. Because Ward agglomerative and spectral clustering lack native out-of-sample prediction, we calculated each discovery cluster’s mean vector in standardised feature space and assigned each alerted test episode to the nearest mean by Euclidean distance. Spectral clustering used a 10-nearest-neighbour affinity graph with K-means discretisation. No clustering model was refitted on test data.

To quantify clinical stratification among alerted patients, we defined the alerted test cohort as final held-out test-set of patients that would trigger an alert at the prespecified probability threshold *p* ≥ 0.08. For each method, we computed cluster-level observed fall incidence with 95% Wilson confidence intervals and summarised outcome separation by (i) incidence range (max–min), (ii) incidence ratio (max/min), and (iii) IQR of incidence across clusters. Larger values indicate greater dispersion of observed incidence across clusters in the alerted cohort. We additionally assessed risk–outcome alignment using Spearman correlation between cluster mean predicted risk and observed incidence, and cluster-level calibration error. For method-level summaries, clusters contributing < 1% of the alerted test cohort were excluded from the separation metrics and reported in the supplement.

**Fig. 5:**
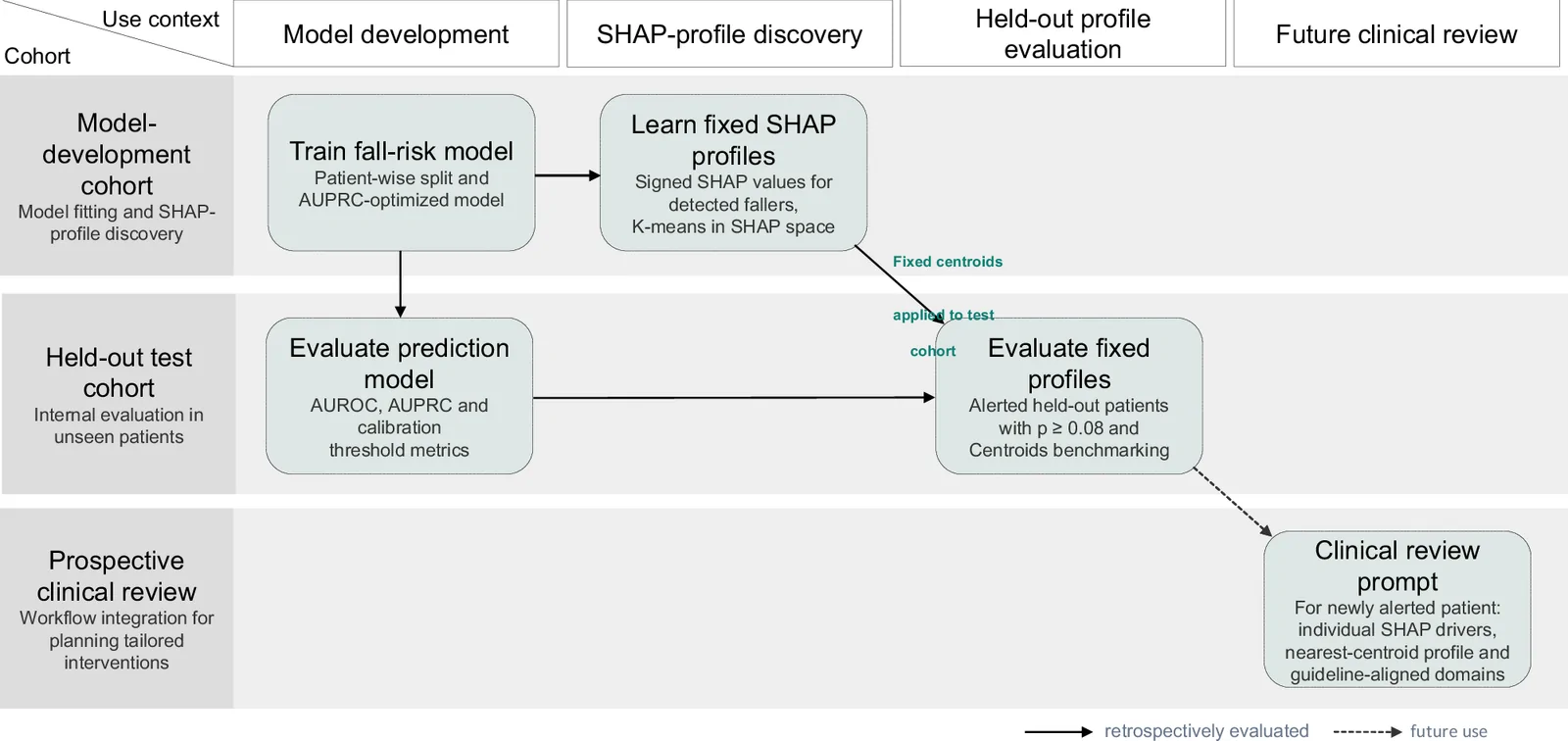
Workflow for model development, profile discovery, profile evaluation, and prospective assignment. The prediction model is trained in the model-development cohort and evaluated in the final held-out test cohort. SHAP profiles are obtained in the discovery cohort and used to learn the *k* = 9 profile centroids. The centroids are then applied to the alerted test cohort to estimate profile sizes, observed incidence, predicted risk, and outcome separation. In future deployment, newly alerted patients can be mapped to the nearest centroid when they fall within the predefined distance threshold; otherwise, they are left unassigned or reviewed using the nearest candidate profiles.

To support future use of the discovered profiles, newly alerted patients can be mapped to the nearest SHAP-profile centroid learned in the discovery cohort. For a newly alerted patient with SHAP vector *S*_*p*_ and cluster centroids *μ*_*k*_, we compute Euclidean distances *d*(*S*_*p*_*, μ*_*k*_) = ||*S*_*p*_−*μ*_*k*_||^2^. The patient is assigned to the nearest centroid k* = argmin *d*(*S*_*p*_, *μ*_*k*_) only if *d*(*S*_*p*_*, μ*_k_*) is no greater than *τ*_1_, where *τ*_1_ is set to the 90th percentile of within-cluster distances estimated in the discovery cohort. Patients exceeding this distance threshold are marked as low-confidence matches and left unassigned; the nearest candidate profiles may still be displayed to support clinical review. This reject option is intended to avoid forcing unstable assignments for patients who do not closely resemble any discovered profile. The overall modelling pipeline is sketched in Fig. 5.

Finally, we used the World Falls Guidelines to map the dominant, cluster-level risk domains to the most plausible intervention domains. First, we identified the dominant SHAP drivers for each cluster. Second, we grouped these drivers into broader fall-prevention domains, such as mobility and transfer impairment, cognition and behaviour, or medication exposure. Third, we mapped these domains to corresponding assessment and intervention domains in the World Falls Guidelines^18^. Additionally, the RNAO falls-prevention guideline was used to distinguish non-modifiable risk markers, such as advanced age or previous falls, from potentially modifiable or intervention-amenable domains, such as impaired gait, balance or mobility^53^. This mapping provides a structured starting point for clinical review, but its effect on fall prevention requires prospective validation.

### Ethics approval and consent to participate

This retrospective study was approved by the Charité Ethics Commission (Approval No. EA2/184/21). Original approval dated 20 August 2021; amendment approved on 2 December 2024 (amendment submitted on 20 November 2024). For this retrospective study, informed consent was waived by the ethics board. All procedures were performed in accordance with the Declaration of Helsinki and its later amendments (or comparable ethical standards). For retrospective analyses of routinely collected data, informed consent was waived by the ethics committee where applicable.

## Supplementary information

Supplementary Information

## Data Availability

The patient-level electronic health record data analysed in this study are not publicly available due to privacy, ethical, and legal restrictions. De-identified data may be made available from the corresponding author on reasonable request and subject to institutional approval, applicable data-protection regulations, and a data-use agreement that prohibits re-identification and onward sharing.

## Code availability

Python was used for data processing, visualization, and modeling. The Python scripts are available from the corresponding author upon reasonable request.
