# Supplementary material for: Explainable SHAP-space fall risk profiling from electronic health records for inpatient prevention planning
Source: Npj Health Syst. 2026 Sep 10;3:90. doi: 10.1038/s44401-026-00138-4 (PMC13562730; doi:10.1038/s44401-026-00138-4)
Supplement: Supplementary file 1 [file 44401_2026_138_MOESM1_ESM.docx]

# Supplementary Information

## Supplementary Table 1: hospital fall-risk assessment benchmark

We evaluated the university hospital’s nursing fall-risk assessment, which categorises patients as ‘0’ (no increased risk of falling), ‘+’ (increased risk), or ‘++’ (significantly increased risk). These analyses are restricted to admissions with an available hospital fall-risk assessment score and complete benchmark variables, which explains the smaller sample size relative to the full analytic cohort. Logistic regression with a 90%/10% train-test split was used to estimate the association between the hospital fall-risk category and documented fall incidents. Supplementary Table 1 reports regression coefficients and model statistics. The predicted fall probability increased monotonically across the three categories: approximately 0.46% for patients scored “0”, 2.57% for patients scored “+”, and 7.00% for patients scored “++”. Pairwise comparisons implied by the fitted model indicate that, relative to patients scored “0”, the odds of a documented fall were approximately 5.65-fold higher for patients scored “+” and 16.13-fold higher for patients scored “++”.

Supplementary Table 1 | Logistic regression benchmark for the university hospital fall-risk assessment. The dependent variable is documented fall incident status, and the predictor is the hospital’s three-level fall-risk assessment category.

| No. Observations: 511,551 LLR p-value: 0.000 | | | | Pseudo R-squ.: 0.07142 Log-Likelihood: -31983. | | |
| --- | --- | --- | --- | --- | --- | --- |
|  | coef | std err | z | P >\|z\| | [0.025 | 0.975] |
| const | -3.8824 | 0.103 | -37.654 | 0.000 | -4.085 | -3.680 |
| 0 | -1.4846 | 0.106 | -13.978 | 0.000 | -1.693 | -1.276 |
| + | 0.2461 | 0.104 | 2.361 | 0.018 | 0.042 | 0.451 |
| ++ | 1.2958 | 0.113 | 11.449 | 0.000 | 1.074 | 1.518 |

# Supplementary Tables 2–3: diagnosis and procedure summaries

Supplementary Table 2 shows the most common procedure groups among fallers and non-fallers, and Supplementary Table 3 shows the most common diagnosis groups.

Supplementary Table 2 | Most common procedure groups among fallers and non-fallers.

| ***Fallen Patients*** | | ***Non-fallen Patients*** | |
| --- | --- | --- | --- |
| **Procedure group** | **Count, n (%)** | **Procedure group** | **Count, n (%)** |
| Computer Tomography CT | 17,326 (16.33) | Computer Tomography CT | 607,914 (15.07) |
| Circulatory Measures | 13,227 (12.47) | Circulatory Measures | 413,399 (10.25) |
| Radiotherapy, nuclear medical therapy, and chemotherapy | 6,260 (5.90) | Radiotherapy, nuclear medical therapy, and chemotherapy | 260,039 (6.45) |
| Patient monitoring | 5,610 (5.29) | Examination of individual body systems | 222,475 (5.51) |
| Treatment for mental and psychosomatic, and behavioural disorders in adults | 5,170 (4.87) | Surgeries on the locomotor organs | 188,950 (4.68) |

Supplementary Table 3 | Most common diagnosis groups for fallers and non-fallers.

| ***Fallen Patients*** | | ***Non-fallen Patients*** | |
| --- | --- | --- | --- |
| **Diagnosis group** | **Count, n (%)** | **Diagnosis group** | **Count, n (%)** |
| Forms of heart disease | 5,339 (5.35) | Potential health risks based on family/personal history | 413,035 (5.36) |
| Metabolic disorders | 5,295 (5.31) | Forms of heart disease | 338,275 (4.39) |
| Potential health risks based on family/personal history | 4,197 (4.21) | Metabolic disorders | 321,760 (4.17) |
| Hypertension | 3,184 (3.19) | Hypertension | 258,783 (3.36) |
| Diseases of the muscles | 2,812 (2.82) | Persons using the health care system for the purpose of specific measures and medical care | 171,177 (2.22) |

# Supplementary Tables 4-5: nursing missingness sensitivity analysis

Supplementary Table 4 | Nursing missingness sensitivity analysis. Model performance is shown for the full EHR model, a model excluding nursing variables, a model retaining only nursing-documentation indicators, and a model using demographics plus nursing-documentation indicators only.

| Model | Features | AUROC | AUPRC | Interpretation |
| --- | --- | --- | --- | --- |
| Full model | 315 | 0.9491 | 0.3644 | Full EHR model including substantive nursing variables |
| No nursing variables | 264 | 0.9437 | 0.2727 | Nursing-care variables removed |
| Non-nursing + nursing-documentation indicators | 315 | 0.9473 | 0.2970 | Substantive nursing values removed; only documentation status retained |
| Nursing-documentation indicators + demographics only | 53 | 0.7918 | 0.0853 | Documentation-process signal without broader EHR content |

The feature-ablation sensitivity models were refit under feature configurations using the same patient-wise split as the primary analysis.

Supplementary Table 5 | Paired bootstrap contrasts for the nursing missingness sensitivity analysis. Differences were estimated using 2,000 paired bootstrap resamples at the patient level.

| Contrast | ΔAUROC | 95% CI | ΔAUPRC | 95% CI | Interpretation |
| --- | --- | --- | --- | --- | --- |
| Non-nursing + nursing-documentation indicators vs. no nursing variables | +0.00358 | 0.00183 to 0.00524 | +0.02426 | 0.01348 to 0.03533 | Incremental documentation-process signal |
| Full model vs. non-nursing + nursing-documentation indicators | +0.00186 | 0.00031 to 0.00336 | +0.06744 | 0.05463 to 0.07982 | Incremental nursing-value signal beyond documentation status |

# Supplementary Tables 6–7 and Supplementary Figure 1: 24-hour landmark analysis

Supplementary Table 6 | Twenty-four-hour admission landmark sensitivity analysis. The model was trained and evaluated at the admission level. Only information available at or before admission plus 24 hours was included. Falls at or before the landmark were excluded because they were not part of the post-landmark prediction target. Confidence intervals were estimated using 2,000 patient-level bootstrap resamples.

| Measure | Value |
| --- | --- |
| Landmark | 24 hours after admission |
| Analysis unit | Admission/case |
| Split unit | Patient |
| Modelled admissions | 594,737 |
| Patients in modelled cohort | 324,308 |
| Post-landmark falls | 6,733 |
| Falls at or before 24 hours excluded | 1,329 |
| Held-out test admissions | 119,022 |
| Held-out test patients | 64,715 |
| Held-out test falls | 1,404 |
| Held-out test prevalence | 1.18% |
| Feature count | 351 |
| AUROC | 0.798 |
| AUROC 95% CI | 0.786–0.809 |
| AUPRC | 0.0529 |
| AUPRC 95% CI | 0.0468–0.0606 |
| No-information AUPRC baseline | 0.0118 |
| Calibration slope | 0.869 |
| Brier score | 0.0114 |
| Sensitivity at p ≥ 0.08 | 10.2% |
| Specificity at p ≥ 0.08 | 98.8% |
| PPV at p ≥ 0.08 | 9.1% |
| NPV at p ≥ 0.08 | 98.9% |

Supplementary Table 7 | Descriptive comparison of the primary full-window patient-level model and the 24-hour admission landmark model. The comparison is descriptive rather than paired because of the difference in the analysis unit, prevalence, and outcome definition.

| Metric | Primary model | 24-hour admission landmark model | Landmark minus primary |
| --- | --- | --- | --- |
| AUROC | 0.9463 | 0.7978 | −0.1485 |
| AUPRC | 0.3568 | 0.0529 | −0.3039 |

Supplementary Fig. 1 | Performance of the 24-hour admission landmark model. This figure shows the precision-recall, receiver operating characteristic, and calibration curves for the held-out test cohort. The precision-recall panel includes the no-information baseline, which corresponds to the test-set prevalence of 1.18%. The ROC curve shows discrimination under the fixed 24-hour prediction horizon. The calibration panel shows the observed fall proportions across the predicted-risk bins relative to the ideal calibration line.

# Supplementary Tables 8-9 and Supplementary Figure 2: Temporal out-of-sample validation

Supplementary Table 8 | Temporal out-of-sample validation. The model was trained on patients indexed from 2016 to 2020, then validated and calibrated using patients from 2021, and finally tested using patients from 2022. In the conservative main analysis (“Temporal validation”), cross-period patients were excluded to avoid leakage across temporal split boundaries. The sensitivity analysis assigned cross-period patients to their earliest eligible temporal split.

| Measure | Temporal validation | Earliest-assignment sensitivity |
| --- | --- | --- |
| Training period | 2016–2020 | 2016–2020 |
| Validation/calibration period | 2021 | 2021 |
| Temporal test period | 2022 | 2022 |
| Cross-period strategy | Exclude | Assign to earliest eligible split |
| Cross-period patients affected | 110,710 | 110,710 |
| Cross-period fallers affected | 2,327 | 2,327 |
| Training patients | 638,618 | 732,970 |
| Validation patients | 91,659 | 108,017 |
| Temporal test patients | 61,186 | 61,186 |
| Temporal test falls | 358 | 358 |
| Temporal test prevalence | 0.59% | 0.59% |
| Feature count | 312 | 312 |
| AUROC | 0.953 | 0.953 |
| AUROC 95% CI | 0.941–0.962 | 0.942–0.962 |
| AUPRC | 0.337 | 0.342 |
| AUPRC 95% CI | 0.286–0.388 | 0.293–0.393 |
| No-information AUPRC baseline | 0.0059 | 0.0059 |
| AUPRC lift over baseline | 57.5× | 58.5× |
| Calibration slope | 0.962 | 0.971 |
| Brier score | 0.00468 | 0.00468 |
| Sensitivity at p ≥ 0.08 | 55.0% | 57.5% |
| Specificity at p ≥ 0.08 | 98.2% | 98.1% |
| PPV at p ≥ 0.08 | 15.0% | 14.9% |
| NPV at p ≥ 0.08 | 99.7% | 99.7% |
| Alerts at p ≥ 0.08 | 1,309 | 1,380 |
| Alert rate at p ≥ 0.08 | 2.14% | 2.26% |

Supplementary Table 9 | Descriptive comparison of random patient-wise split and temporal out-of-sample validation.

| Metric | Random patient-wise split | Conservative temporal 2022 test | Earliest-assignment sensitivity |
| --- | --- | --- | --- |
| Test patients | 186,421 | 61,186 | 61,186 |
| Test falls | 2,164 | 358 | 358 |
| Test prevalence | 1.16% | 0.59% | 0.59% |
| AUROC | 0.946 | 0.953 | 0.953 |
| AUPRC | 0.357 | 0.337 | 0.342 |
| AUPRC baseline | 0.0116 | 0.0059 | 0.0059 |
| AUPRC lift over baseline | 30.7× | 57.5× | 58.5× |

Note: The temporal validation uses a different split strategy and excludes cross-period patients conservatively. Since AUPRC is prevalence-sensitive, the lower AUPRC in the 2022 temporal test set should be interpreted alongside the lower fall prevalence in 2022 and the lift over the baseline, which is normalised with regard to prevalence.

Supplementary Fig. 2 | Temporal out-of-sample validation performance in the 2022 test set. Precision-recall, receiver-operating-characteristic, and calibration curves for the temporal test cohort. The model was trained using patients from 2016 to 2020, validated and calibrated using patients from 2021, and tested using patients from 2022. The precision-recall panel shows an AUPRC of 0.337 against the baseline, which corresponds to the fall 2022 prevalence of 0.59%. The ROC panel shows preserved discrimination over time, with an AUROC of 0.953. The calibration panel shows risk-decile calibration with Wilson confidence intervals, Brier score 0.00468, and calibration slope 0.962.

## Supplementary Fig. 3: Global SHAP explanations

For the L3 model chosen, we examined the aggregated local feature importances using SHAP. Supplementary Fig. 3 is a SHAP summary plot for the top features contributing to fall risk. Each point represents a patient; points are plotted horizontally by SHAP value, i.e., the impact on model output, and are coloured by the feature value. The strongest global driver was age, followed by the documented fall history within the previous 12 months, and indicators of care complexity. Procedure-code groups such as supplementary care information, complex treatment, and computed tomography contributed to predicted fall risk in subsets of patients; nursing-derived variables, including cognition impairment, transfer impairment, walking impairment, and mobility-related scores, were also among the top-ranked predictors.

Supplementary Fig. 3 | SHAP summary plot of top 20 features for fall risk prediction. The features are ranked by mean absolute SHAP value, i.e. the largest average magnitude of contribution to the model output, regardless of direction. Points to the right indicate that the feature increases fall risk, to the left decreases risk. Colour indicates the feature value, i.e., red for high, blue for low. For continuous features like age, red means older age. For binary features (procedures, diagnoses), red indicates presence of that code, blue indicates absence. Grey colour indicates categorical features.

## Supplementary Tables 10-11: k-sensitivity and cluster-level diagnostics

Supplementary Table 10 | k-sensitivity diagnostics for SHAP-space K-means. Candidate k-solutions were fitted in the profile-discovery cohort. Internal clustering diagnostics and minimum discovery-cluster size were computed in the discovery cohort; outcome-separation diagnostics were computed after applying the fixed centroids to the alerted held-out test cohort.

| k | Silhouette | Davies–Bouldin | Gap statistic | ARI mean (95% interval) | Min discovery n (%) | Alerted test: effective clusters | Alerted test: observed-incidence range | Alerted test: predicted-observed Spearman |
| --- | --- | --- | --- | --- | --- | --- | --- | --- |
| 4 | 0.168 | 1.705 | 2.293 | 0.784 (0.589–0.977) | 372 (12.3%) | 4 | 0.103 | 0.800 |
| 5 | 0.182 | 1.613 | 2.356 | 0.916 (0.748–0.980) | 110 (3.6%) | 5 | 0.103 | 0.800 |
| 6 | 0.162 | 1.628 | 2.395 | 0.834 (0.615–0.982) | 110 (3.6%) | 6 | 0.106 | 0.829 |
| 7 | 0.167 | 1.793 | 2.437 | 0.926 (0.708–0.981) | 105 (3.5%) | 7 | 0.130 | 0.929 |
| 8 | 0.158 | 1.858 | 2.470 | 0.901 (0.700–0.978) | 103 (3.4%) | 8 | 0.337 | 0.857 |
| 9 | 0.155 | 1.774 | 2.501 | 0.856 (0.687–0.965) | 103 (3.4%) | 9 | 0.325 | 0.783 |
| 10 | 0.165 | 1.790 | 2.532 | 0.867 (0.741–0.956) | 103 (3.4%) | 10 | 0.333 | 0.891 |
| 11 | 0.169 | 1.711 | 2.563 | 0.903 (0.809–0.964) | 76 (2.5%) | 11 | 0.306 | 0.909 |
| 12 | 0.178 | 1.687 | 2.590 | 0.900 (0.808–0.952) | 75 (2.5%) | 12 | 0.320 | 0.888 |
| 13 | 0.182 | 1.701 | 2.607 | 0.837 (0.734–0.934) | 76 (2.5%) | 12 | 0.197 | 0.860 |
| 14 | 0.186 | 1.823 | 2.624 | 0.792 (0.688–0.910) | 68 (2.3%) | 12 | 0.179 | 0.734 |

Note: The discovery cohort comprised detected fallers from the development cohort and was used to learn the SHAP-profile centroids. Silhouette score, Davies–Bouldin index, gap statistic, bootstrap adjusted Rand index, and minimum discovery-cluster size were computed in this discovery cohort and used to assess geometric coherence and stability. Effective alerted test clusters, observed-incidence range, and predicted-observed Spearman correlation were computed only after applying the fixed centroids to the alerted held-out test cohort and were used as evaluation diagnostics. Higher values indicate better performance for silhouette score, gap statistic, bootstrap adjusted Rand index, observed-incidence range, and predicted-observed Spearman correlation; lower values indicate better performance for the Davies–Bouldin index. These diagnostics support k = 9 as a viable solution, but not as a uniquely optimal mathematical solution.

Supplementary Table 11 | Full cluster-level diagnostics for the *k* = 9 SHAP-based risk-profile solution. Discovery-cohort columns describe the discovery cohort used to learn the centroids. Alerted-test-cohort columns describe the held-out test-set patients with predicted fall risk *p* ≥ 0.08 after applying the centroids.

| **Cluster ID** | **Profile label** | **Discovery cohort, n** | **Discovery cohort, %** | **Alerted test cohort, n** | **Alerted test cohort, %** | **Observed falls, n** | **Observed incidence** | **95% CI lower** | **95% CI upper** | **Mean predicted risk** | **Predicted risk, SD** | **Median age, years** | **Age IQR, years** | **Male, %** | **Nearest profile ID** | **Nearest profile label** | **Nearest-centroid distance** |
| --- | --- | --- | --- | --- | --- | --- | --- | --- | --- | --- | --- | --- | --- | --- | --- | --- | --- |
| 0 | C1 | 352 | 11.67 | 1466 | 16.04 | 203 | 0.14 | 0.12 | 0.16 | 0.15 | 0.12 | 75 | 22 | 58.12 | 4 | C5 | 0.050 |
| 1 | C2 | 276 | 9.15 | 760 | 8.32 | 143 | 0.19 | 0.16 | 0.22 | 0.18 | 0.15 | 75 | 21 | 57.11 | 4 | C5 | 0.061 |
| 2 | C3 | 660 | 21.88 | 3257 | 35.64 | 526 | 0.16 | 0.15 | 0.17 | 0.16 | 0.10 | 78 | 19 | 51.64 | 6 | C7 | 0.042 |
| 3 | C4 | 171 | 5.67 | 140 | 1.53 | 63 | 0.45 | 0.37 | 0.53 | 0.37 | 0.26 | 73 | 21 | 54.29 | 1 | C2 | 0.062 |
| 4 | C5 | 276 | 9.15 | 760 | 8.32 | 95 | 0.13 | 0.10 | 0.15 | 0.17 | 0.16 | 71 | 25 | 60.34 | 8 | C9 | 0.041 |
| 5 | C6 | 103 | 3.42 | 113 | 1.24 | 28 | 0.25 | 0.18 | 0.33 | 0.28 | 0.22 | 75 | 27 | 48.67 | 2 | C3 | 0.081 |
| 6 | C7 | 333 | 11.04 | 657 | 7.19 | 101 | 0.15 | 0.13 | 0.18 | 0.18 | 0.17 | 72 | 32 | 44.14 | 2 | C3 | 0.042 |
| 7 | C8 | 280 | 9.28 | 1394 | 15.25 | 190 | 0.14 | 0.12 | 0.16 | 0.15 | 0.12 | 70 | 23 | 61.91 | 4 | C5 | 0.049 |
| 8 | C9 | 565 | 18.73 | 592 | 6.48 | 101 | 0.17 | 0.14 | 0.20 | 0.23 | 0.21 | 66 | 35 | 52.03 | 4 | C5 | 0.041 |

## Supplementary Tables 12 and Supplementary Figures 4-5: lower-k merge diagnostics and cluster-level validation

Supplementary Table 12 | Lower-k merge diagnostics for adjacent k = 9 SHAP-based risk profiles. Centroid distance was calculated in raw signed SHAP space. Incidence and predicted-risk differences are absolute differences in percentage points in the alerted test cohort. Dominant drivers are mean signed SHAP attributions; positive values increase predicted fall risk and negative values decrease predicted fall risk relative to the model baseline. Lower-k behaviour indicates whether the two k = 9 profiles were merged or remained separate in the k = 7 and k = 8 solutions.

| Profile pair | Centroid distance | Lower-k behaviour | Observed-incidence difference | Predicted-risk difference | Dominant drivers, profile A | Dominant drivers, profile B | Interpretation |
| --- | --- | --- | --- | --- | --- | --- | --- |
| C5 / C9 | 0.041 | k = 7: separate; k = 8: separate | 4.6 pp | 5.5 pp | C5: Computed tomography; care-episode/movement marker; prior-fall documentation; circulatory measures | C9: Examination/clarification; prior-fall documentation; complex treatment; hypertension | Profiles remained separate in both lower-k solutions and differed in predicted risk. C9 had weaker attribution magnitudes and should not be interpreted as a direct clinical counterpart of C5. |
| C3 / C7 | 0.042 | k = 7: separate; k = 8: separate | 0.8 pp | 2.0 pp | C3: Supplementary care information; computed tomography; early rehabilitation/physical therapy; head injury | C7: Mental, psychosomatic and behavioural disorders; Supplementary care information; early rehabilitation/physical therapy; complex treatment | Profiles had similar observed incidence but remained separate in lower-k solutions. Their shared care-complexity signal suggests related profiles, but C3 was more CT/head-injury oriented, whereas C7 was more behavioural/mental-health oriented. |
| C5 / C8 | 0.049 | k = 7: separate; k = 8: separate | 1.1 pp | 1.8 pp | C5: Computed tomography; care-episode/movement marker; prior-fall documentation; circulatory measures | C8: Complex treatment; computed tomography; radiotherapy/nuclear medicine therapy/chemotherapy; care-episode/movement marker | Profiles had similar risk levels but remained separate under lower-k solutions. The distinction reflects different attribution pathways: CT/prior-fall/circulatory signals in C5 versus complex-treatment and oncology-associated signals in C8. |
| C1 / C5 | 0.050 | k = 7: separate; k = 8: separate | 1.3 pp | 2.4 pp | C1: Early rehabilitation/physical therapy; computed tomography; complex treatment; examination/clarification | C5: Computed tomography; care-episode/movement marker; prior-fall documentation; circulatory measures | Profiles had similar observed risk but remained separate in lower-k solutions. C1 was rehabilitation/functional-care oriented, whereas C5 was CT/prior-fall/circulatory oriented. |
| C1 / C8 | 0.050 | k = 7: merged; k = 8: merged | 0.2 pp | 0.6 pp | C1: Early rehabilitation/physical therapy; computed tomography; complex treatment; examination/clarification | C8: Complex treatment; computed tomography; radiotherapy/nuclear medicine therapy/chemotherapy; care-episode/movement marker | C1 and C8 were the strongest merge candidates. They had nearly identical observed and predicted risk and merged under lower-k solutions. Because the overall k = 9 solution met the prespecified viability criteria, we retained the k = 9 profile set, but interpret C1 and C8 as related profiles. C1 was more rehabilitation/functional-care oriented, whereas C8 was more complex-treatment and oncology-associated. |

Supplementary Fig. 4 | k-sensitivity diagnostics for SHAP-space K-means. Internal clustering metrics were evaluated for k = 4 through k = 14. The figure shows silhouette score, Davies–Bouldin index, Calinski–Harabasz score, and gap statistic. The diagnostics show that k = 9 was viable but not uniquely optimal; therefore, the selected solution is interpreted as a clinically useful resolution rather than a mathematical optimum.

Supplementary Fig. 5 | Bootstrap stability of SHAP-space K-means across k. Stability was assessed using 100 bootstrap resamples and the out-of-bag adjusted Rand index. The k = 9 solution had mean adjusted Rand index 0.856 with a 95% interval of 0.687–0.965, supporting reproducibility while indicating some hierarchical instability among adjacent profiles.

## Supplementary Table 13: comparator cluster-level outcomes in the alerted test cohort

For each clustering method and each resulting cluster, we report cluster size, number of falls, observed fall incidence with 95% Wilson confidence interval, and mean predicted fall risk after applying the clustering solution to the alerted test cohort. Small clusters are shown here for transparency, even when they were excluded from method-level separation metrics in Table 3 because they contributed <1% of the alerted test cohort.

Supplementary Table 13 | Cluster-level outcomes in the alerted test cohort, defined as held-out test-set patients with predicted fall risk p≥0.08.

| **Method** | **Cluster** | **Alerted test *n*** | **Alerted test %** | **Falls, n** | **Observed incidence** | **Observed incidence 95% CI lower** | **Observed incidence 95% CI upper** | **Mean predicted risk** | **Predicted risk, SD** |
| --- | --- | --- | --- | --- | --- | --- | --- | --- | --- |
| SHAP_KMeans | 0 | 1466 | 16.041 | 203 | 0.138 | 0.122 | 0.157 | 0.146 | 0.116 |
| SHAP_KMeans | 1 | 760 | 8.316 | 143 | 0.188 | 0.162 | 0.217 | 0.176 | 0.150 |
| SHAP_KMeans | 2 | 3257 | 35.638 | 526 | 0.161 | 0.149 | 0.175 | 0.164 | 0.096 |
| SHAP_KMeans | 3 | 140 | 1.532 | 63 | 0.450 | 0.370 | 0.533 | 0.366 | 0.263 |
| SHAP_KMeans | 4 | 760 | 8.316 | 95 | 0.125 | 0.103 | 0.150 | 0.170 | 0.162 |
| SHAP_KMeans | 5 | 113 | 1.236 | 28 | 0.248 | 0.177 | 0.335 | 0.278 | 0.219 |
| SHAP_KMeans | 6 | 657 | 7.189 | 101 | 0.154 | 0.128 | 0.183 | 0.184 | 0.169 |
| SHAP_KMeans | 7 | 1394 | 15.253 | 190 | 0.136 | 0.119 | 0.155 | 0.152 | 0.118 |
| SHAP_KMeans | 8 | 592 | 6.478 | 101 | 0.171 | 0.142 | 0.203 | 0.225 | 0.209 |
| RAW_KMeans | 0 | 2566 | 28.077 | 384 | 0.150 | 0.136 | 0.164 | 0.149 | 0.120 |
| RAW_KMeans | 1 | 577 | 6.314 | 90 | 0.156 | 0.129 | 0.188 | 0.160 | 0.095 |
| RAW_KMeans | 2 | 1047 | 11.456 | 166 | 0.159 | 0.138 | 0.182 | 0.156 | 0.094 |
| RAW_KMeans | 3 | 1062 | 11.621 | 170 | 0.160 | 0.139 | 0.183 | 0.184 | 0.166 |
| RAW_KMeans | 4 | 2138 | 23.394 | 286 | 0.134 | 0.120 | 0.149 | 0.159 | 0.088 |
| RAW_KMeans | 5 | 37 | 0.405 | 1 | 0.027 | 0.005 | 0.138 | 0.153 | 0.121 |
| RAW_KMeans | 6 | 1408 | 15.406 | 292 | 0.207 | 0.187 | 0.229 | 0.227 | 0.220 |
| RAW_KMeans | 7 | 109 | 1.193 | 18 | 0.165 | 0.107 | 0.246 | 0.159 | 0.096 |
| RAW_KMeans | 8 | 195 | 2.134 | 43 | 0.221 | 0.168 | 0.284 | 0.234 | 0.097 |
| RAW_GMM | 0 | 4801 | 52.533 | 693 | 0.144 | 0.135 | 0.155 | 0.156 | 0.104 |
| RAW_GMM | 1 | 28 | 0.306 | 12 | 0.429 | 0.265 | 0.609 | 0.328 | 0.252 |
| RAW_GMM | 2 | 18 | 0.197 | 18 | 1.000 | 0.824 | 1.000 | 0.648 | 0.041 |
| RAW_GMM | 3 | 395 | 4.322 | 101 | 0.256 | 0.215 | 0.301 | 0.248 | 0.227 |
| RAW_GMM | 4 | 41 | 0.449 | 24 | 0.585 | 0.434 | 0.722 | 0.436 | 0.274 |
| RAW_GMM | 5 | 42 | 0.460 | 1 | 0.024 | 0.004 | 0.123 | 0.141 | 0.113 |
| RAW_GMM | 6 | 3773 | 41.285 | 595 | 0.158 | 0.146 | 0.170 | 0.175 | 0.152 |
| RAW_GMM | 7 | 28 | 0.306 | 5 | 0.179 | 0.079 | 0.356 | 0.193 | 0.145 |
| RAW_GMM | 8 | 13 | 0.142 | 1 | 0.077 | 0.014 | 0.333 | 0.143 | 0.053 |
| RAW_Ward | 0 | 1824 | 19.958 | 357 | 0.196 | 0.178 | 0.215 | 0.226 | 0.214 |
| RAW_Ward | 1 | 1890 | 20.681 | 304 | 0.161 | 0.145 | 0.178 | 0.165 | 0.092 |
| RAW_Ward | 2 | 151 | 1.652 | 18 | 0.119 | 0.077 | 0.181 | 0.153 | 0.087 |
| RAW_Ward | 3 | 13 | 0.142 | 0 | 0.000 | 0.000 | 0.228 | 0.114 | 0.037 |
| RAW_Ward | 4 | 2480 | 27.136 | 410 | 0.165 | 0.151 | 0.180 | 0.155 | 0.132 |
| RAW_Ward | 5 | 2340 | 25.605 | 317 | 0.135 | 0.122 | 0.150 | 0.153 | 0.091 |
| RAW_Ward | 6 | 6 | 0.066 | 0 | 0.000 | 0.000 | 0.390 | 0.109 | 0.040 |
| RAW_Ward | 7 | 415 | 4.541 | 44 | 0.106 | 0.080 | 0.139 | 0.154 | 0.105 |
| RAW_Ward | 8 | 20 | 0.219 | 0 | 0.000 | 0.000 | 0.161 | 0.179 | 0.154 |
| RAW_Spectral | 0 | 3123 | 34.172 | 577 | 0.185 | 0.172 | 0.199 | 0.194 | 0.189 |
| RAW_Spectral | 1 | 42 | 0.460 | 4 | 0.095 | 0.038 | 0.221 | 0.187 | 0.158 |
| RAW_Spectral | 2 | 174 | 1.904 | 23 | 0.132 | 0.090 | 0.191 | 0.164 | 0.125 |
| RAW_Spectral | 3 | 104 | 1.138 | 15 | 0.144 | 0.089 | 0.224 | 0.154 | 0.095 |
| RAW_Spectral | 4 | 280 | 3.064 | 52 | 0.186 | 0.145 | 0.235 | 0.174 | 0.120 |
| RAW_Spectral | 5 | 79 | 0.864 | 14 | 0.177 | 0.109 | 0.276 | 0.155 | 0.100 |
| RAW_Spectral | 6 | 123 | 1.346 | 14 | 0.114 | 0.069 | 0.182 | 0.181 | 0.137 |
| RAW_Spectral | 7 | 330 | 3.611 | 39 | 0.118 | 0.088 | 0.157 | 0.172 | 0.128 |
| RAW_Spectral | 8 | 4884 | 53.441 | 712 | 0.146 | 0.136 | 0.156 | 0.156 | 0.096 |
| PCA95_KMeans | 0 | 477 | 5.219 | 68 | 0.143 | 0.114 | 0.177 | 0.177 | 0.129 |
| PCA95_KMeans | 1 | 3293 | 36.032 | 472 | 0.143 | 0.132 | 0.156 | 0.146 | 0.110 |
| PCA95_KMeans | 2 | 874 | 9.563 | 149 | 0.170 | 0.147 | 0.197 | 0.194 | 0.186 |
| PCA95_KMeans | 3 | 1 | 0.011 | 0 | 0.000 | 0.000 | 0.793 | 0.152 | NA |
| PCA95_KMeans | 4 | 2 | 0.022 | 0 | 0.000 | 0.000 | 0.658 | 0.093 | 0.015 |
| PCA95_KMeans | 5 | 21 | 0.230 | 0 | 0.000 | 0.000 | 0.155 | 0.129 | 0.066 |
| PCA95_KMeans | 6 | 882 | 9.651 | 169 | 0.192 | 0.167 | 0.219 | 0.170 | 0.097 |
| PCA95_KMeans | 7 | 2226 | 24.357 | 308 | 0.138 | 0.125 | 0.153 | 0.165 | 0.089 |
| PCA95_KMeans | 8 | 1363 | 14.914 | 284 | 0.208 | 0.188 | 0.231 | 0.225 | 0.219 |

**Notes**: Observed incidence is computed on the alerted test cohort, defined as held-out test-set patients with predicted fall risk p ≥ 0.08. Wilson CIs are reported for binomial proportions. Mean predicted risk is the model’s mean predicted probability within each cluster.
